# m^1^A and m^6^A RNA Methylations as Druggable Targets in Cancer

**DOI:** 10.3390/ph19070990

**Published:** 2026-06-25

**Authors:** Yasemin Gazaloğlu, Buket Sağlam-Şen, Bünyamin Akgül

**Affiliations:** Noncoding RNA Laboratory, Department of Molecular Biology and Genetics, İzmir Institute of Technology, 35430 Izmir, Türkiye; yasemingazaloglu@iyte.edu.tr (Y.G.); buketsaglam@iyte.edu.tr (B.S.-Ş.)

**Keywords:** epitranscriptomics, RNA modifications, adenosine methylations, targeted cancer therapy, small molecule inhibitors, proteolysis-targeting chimeras (PROTACs), translational reprogramming

## Abstract

Epitranscriptomic modifications, particularly RNA methylations, have emerged as regulators of gene expression, with their dysregulation acting as a key factor in tumorigenesis and metastatic progression. This review evaluates the therapeutic landscapes of N^6^-methyladenosine (m^6^A) and N^1^-methyladenosine (m^1^A) modifications in cancer. While the m^6^A machinery predominantly dictates mRNA turnover and stability, the m^1^A network is uniquely positioned to drive translational reprogramming, allowing malignant cells to endure severe microenvironmental stress and evade cell death. Despite positional and chemical differences, these modifications exhibit profound epitranscriptomic crosstalk through shared regulatory proteins. Here, we comprehensively analyze current pharmacological strategies targeting the m^6^A axis, highlighting the transition from classical small-molecule inhibitors of regulatory proteins of these methylations, such as methyltransferase-like 3 (METTL3), fat mass and obesity-associated protein (FTO), and AlkB homolog 5 (ALKBH5), to the novel event-driven approach of proteolysis-targeting chimeras (PROTACs). Furthermore, we assess the emerging therapeutic potential of the m^1^A regulatory machinery, positioning tRNA methyltransferase 6/61A (TRMT6/61A) writers and AlkB homolog 1 to 3 (ALKBH1-3) erasers as promising therapeutic targets. Finally, we discuss clinical successes and current translational obstacles, including off-target toxicity, pharmacokinetic limitations, and epitranscriptomic escape, emphasizing that site-specific modulation and smart precision therapies will dictate the future of oncology.

## 1. Introduction

Epitranscriptomics is an evolving field of RNA biology focused on biochemical modifications of RNA that regulate gene expression without altering the RNA sequence [1]. Epigenetics involves mitotically stable modifications to DNA and histone proteins. These marks primarily regulate the transcription and transcriptional availability of a gene [2]. In contrast, epitranscriptomics acts as a distinct, highly dynamic regulatory layer. It focuses on the modifications of RNA, fine-tuning gene expression both co- and post-transcriptionally [3]. Consequently, while epigenetic marks dictate DNA transcription, epitranscriptomic modifications govern the subsequent fate of RNA transcripts [4]. With over 170 distinct modifications mapped across various RNA types to date, it is now evident that these dynamic marks act as regulators of gene expression networks [5].

This vast epitranscriptomic repertoire encompasses a diverse array of biochemical alterations, including RNA editing events such as adenosine-to-inosine (A-to-I) conversion, base isomerizations like pseudouridylation (ψ), and acetylations like N^4^-acetylcytidine (ac^4^C). Among these diverse marks, RNA methylations constitute a prevalent and therapeutically actionable regulatory layer. Within this extensive RNA methylome, modifications such as N^6^-methyladenosine (m^6^A), 5-methylcytosine (m^5^C) and N^1^-methyladenosine (m^1^A) stand out as the most prominent and extensively characterized regulatory marks [5]. The epitranscriptomic machinery directly shapes cellular fate by governing RNA stability [6], splicing [7], localization [8], and translational output [9]. Consequently, the aberrant regulation of these modification networks serves as a driving force in the pathology of numerous disorders, most notably cancer. Specifically, the altered expression or dysfunction of the core epitranscriptomic enzymes, commonly classified as writer, eraser and readers, disrupt physiological homeostasis and shifts the cellular balance toward oncogenesis [10]. In tumorigenesis, dysregulated RNA modifications reprogram cellular networks to fuel uncontrolled proliferation, apoptotic resistance, and metastatic dissemination [10,11]. Particularly, the interplay between RNA methylation and programmed cell death has emerged as a crucial node in determining tumor survival and advancing RNA-targeted therapeutics [12]. Beyond classical protein-RNA interactions, emerging perspectives indicate that these epitranscriptomic marks execute their regulatory functions by fundamentally altering the structural topology and biophysical properties of targeted RNAs, thereby orchestrating dynamic responses under stress [13]. Recognizing that these complex survival mechanisms are heavily orchestrated by dominant methylations networks, a comprehensive understanding of specific epitranscriptomic marks, particularly m^6^A and m^1^A, is crucial. Consequently, elucidating their precise biological roles and pharmacological targetability provides a fundamental framework for advancing precision oncology [14]. Despite this clinical promise, the field faces significant methodological hurdles. Current biochemical RNA methylation detection technologies provide quantitative, single-base resolution for some methylation marks. However, the existing analytical limitations create inherent challenges in distinguishing between dynamically regulated functional marks and static, structural modifications [15]. In addition to sequencing-based technologies, biophysical approaches are currently being explored as alternative tools. Specifically, Fourier-transform infrared (FT-IR) spectroscopy offers a method to monitor global RNA methylation dynamics and methylation-induced structural alterations [13,16].

While the pharmacological targeting of m^6^A machinery has rapidly advanced into clinical evaluation, the therapeutic potential of the m^1^A regulatory network and the intricate epitranscriptomic crosstalk between these two modifications remain largely unexplored, representing a critical gap in current oncological research. Therefore, this review aims to evaluate the therapeutic landscape of m^1^A and m^6^A modifications in cancer. Furthermore, it analyzes the clinical successes and limitations of m^1^A and m^6^A-targeted therapies, ultimately positioning the m^1^A regulatory network as the next critical frontier for targeted cancer treatment.

## 2. m^1^A and m^6^A RNA Methylations

### 2.1. Overview of m^1^A and m^6^A RNA Methylations

Adenine methylations represent a class of covalent RNA modifications, primarily involving the addition of a methyl group to the N^6^ and N^1^ nitrogen positions of the purine ring. As the best characterized RNA modification, m^6^A involves the covalent addition of a methyl group to the nitrogen atom at the N^6^ position of the adenine base. It is the most abundant internal modification in eukaryotic mRNA [17], while also being highly prevalent in rRNA, tRNA, and various non-coding RNAs. Topologically, m^6^A is highly enriched particularly in the 3’ untranslated regions (3’ UTRs) and near stop codons, strictly localized within the RRACH consensus motif (R = A/G, H = A/C/U) [18,19]. As a biologically dynamic and reversible modification, m^6^A is tightly regulated at the cellular level. This regulation is orchestrated by a specific protein machinery: writer proteins (methyltransferase complexes), eraser proteins (demethylase enzymes), and reader proteins that recognize and bind to the m^6^A mark. Together, these protein networks govern key processes of RNA metabolism, including mRNA stability [6], alternative splicing [7], nuclear export [8], and translational efficiency [9]. In contrast, m^1^A is formed by the covalent addition of a methyl group to the N^1^ position of the adenine base [20]. Under physiological pH conditions, m^1^A alters the protonation of the base, resulting in a positive electrostatic charge [21]. This chemical modification alters the hydrogen bonding capacity of the adenine ring, thereby disrupting canonical A:U Watson–Crick base pairing in favour of Hoogsteen base pairs. This structural perturbation can lead to nucleoside instability and localized disruption of RNA secondary and tertiary structures [22]. m^1^A is most abundant in tRNAs. It is also found in rRNA, ncRNAs and mitochondrial transcripts, though it has been identified in mRNAs at lower stoichiometric levels [23]. The evolutionarily conserved m^1^A modification is found in bacteria, archaea, and eukaryotes, particularly at nucleotide positions 9, 14, 16, 22, 57, and 58 of tRNAs [24].

Among these, the most prevalent m^1^A modification has been identified at position 58 (m^1^A58) within the T-loop region of tRNAs. m^1^A58 modification is paramount for ensuring proper structural folding and stability of the initiator methionyl-tRNA, thus serving as a critical determinant of translation efficiency [25,26].

Similar to m^6^A moieties, the functional landscape of m^1^A is dynamically controlled by its own specific regulatory machinery. The deposition of m^1^A is primarily catalysed by *tRNA* methyltransferase (TRMT) complexes. The TRMT6/TRMT61A complex serves as the primary regulator responsible for m^1^A58 formation in cytosolic tRNAs and a subset of mRNAs, while TRMT61B and TRMT10C predominantly mediate m^1^A methylation in mitochondrial transcripts [23,27,28,29]. The removal of m^1^A is executed by the erasers, such as fat mass and obesity-associated protein (FTO), ALKBH1, and ALKBH3, which belong to the AlkB family of dioxygenases and execute oxidative demethylation [30,31,32]. Interestingly, the downstream functional elements of the m^1^A machinery, members of the YT521-B homolog (YTH) domain-containing family, such as YTHDF1-3 and YTHDC1, have been identified to cross-react and bind m^1^A-modified transcripts [33]. Recently, the dysregulation of this precise m^1^A axis, particularly the overexpression of the TRMT6/TRMT61A complex and the ALKBH3 eraser, has emerged as a pivotal driver in several cancer progression such as lung squamous cell carcinoma and cervical cancer, respectively [34,35]. Specifically, this aberrant regulation fuels cancer cell survival, tumor microenvironment and robust resistance against cellular stress [36].

### 2.2. Biological and Mechanistic Comparison of m^1^A and m^6^A

This m^6^A modification is a dynamic and reversible epitranscriptomic regulation regulated by writer, eraser, and reader proteins. Writer proteins form a methyltransferase complex that adds a methyl group to the N^6^ amino group in mRNA. In this complex, methyltransferase-like 3 (METTL3) protein forms a heterodimer with methyltransferase-like 14 (METTL14) [37]. Within this core, METTL3, as the catalytic enzyme, catalyses the deposition of the methyl group from the S-adenosylmethionine (SAM) binding site to the N^6^ position via the DPPW (Asp-Pro-Pro-Trp) motif in its active site [38], while METTL14 is essential for RNA-substrate binding and stabilizing the heterodimer structure [39]. This methyltransferase complex is accompanied by various cofactor proteins, such as Wilms tumor 1-associated protein (WTAP), vir-like m^6^A methyltransferase-associated (VIRMA or KIAA1429), RNA-binding motif protein 15 (RBM15), and RBM15B paralog. WTAP ensures the binding and stability of the complex to nuclear speckles, while VIRMA facilitates the enrichment of m^6^A in the stop codon area and 3’ UTR of mRNA. Moreover, RBM15 is involved in the placement of m^6^A onto specific RNA transcripts or onto uracil-enriched RNA regions [40,41,42].

m^6^A modification is selectively removed from RNA by eraser proteins. In this process, m^6^A demethylation is catalysed by the iron (Fe(II)) and α-ketoglutarate (α-KG) dependent dioxygenase enzymes FTO (ALKBH9) and AlkB Homolog 5 (ALKBH5) proteins via two-step or one-step oxidative reactions respectively [43,44,45]. FTO is capable of demethylating m^6^A residues in both nuclear and cytoplasmic mRNAs. On the other hand, ALKBH5 is predominantly localized in the nucleus and primarily targets m^6^A-modified mRNAs located in the nuclear speckles. Therefore, the target selection by FTO and ALKBH5 is primarily dictated by subcellular localization of these proteins [32,43].

Reader proteins recognize the m^6^A residues directly or indirectly. Readers harboring a YTH domain, such as YTHDF1-3 and YTHDC1-2, directly bind to the m^6^A moiety in RNA. They can facilitate alternative splicing, mRNA deadenylation, and degradation [7,39]. Insulin-like growth factor-2 mRNA-binding proteins (IGF2BP1-3) regulate mRNA stability and translation by specifically recognizing m^6^A in the GG(m^6^A)C sequence in mRNA [46]. Indirect m^6^A readers recognize their targets through the “m^6^A-switch” mechanism, whereby the secondary structure of RNA is altered upon methylation. HNRNPG and HNRNPC, from the heterogeneous nuclear ribonucleoprotein (HNRNP) protein family, are a classical example of indirect readers, involved in regulating pre-mRNA processing and alternative splicing [47,48].

m^6^A is an epitranscriptomic modification that can occur on protein-coding and non-coding RNAs, playing a significant role in regulating the biological processes of cancer. Studies have shown that m^6^A-modified transcripts can act as both oncogenes and tumor suppressors in cancer development [14]. Alterations in m^6^A regulatory proteins, such as writer, eraser, and reader proteins, have been shown to modulate carcinogenesis, tumor development, and progression in various cancer types, including breast cancer, cervical cancer, prostate cancer, pancreatic cancer, and lung cancer. These alterations can regulate fundamental tumor processes in cancer cells, such as cell proliferation, cell differentiation, epithelial–mesenchymal transition (EMT), migration, invasion, and metastasis. Therefore, methylated transcripts are considered potential biomarkers and therapeutic targets for cancer diagnosis, prognosis, and treatment. [10,11]. Indeed, the dynamic reprogramming of m^6^A regulators not only drives fundamental processes like cell cycle progression [49] but also serves as a indicator of stress adaptation and therapeutic resistance in clinical oncology [50,51].

The functional divergence of m^1^A from m^6^A is most evident in its profound impact on translatome regulation. Rather than predominantly regulating mRNA turnover, m^1^A exerts its oncogenic influence primarily by modulating the translational machinery itself [10]. At the core of this mechanism is m^1^A modifications on tRNAs, particularly the initiator methionyl-tRNA (tRNAi-Met). The TRMT6/TRMT61A-mediated methylation is essential for maintaining structural stability and regulating the dynamic landscape of both cytosolic and mitochondrial transcriptomes [23]. Furthermore, the dynamic erasure of m^1^A on tRNAs (m^1^A58) by specific demethylases, such as ALKBH1, directly dictates global translation rates, thereby establishing the baseline for cellular translation capacity [31].

In the context of cancer, this epitranscriptomic control is hijacked to drive translational reprogramming. As solid tumors rapidly outgrow their blood supply, cancer cells are subjected to severe microenvironmental stresses, including hypoxia, nutrient deprivation, and oxidative stress. While such conditions typically trigger a global shutdown of protein synthesis to conserve cellular energy, malignant cells paradoxically upregulate the m^1^A machinery to selectively translate stress-response and pro-survival transcripts. Elevated m^1^A levels maintain the stability of specific tRNAs and optimize ribosome engagement, enabling the continuous translation of key oncogenes (such as *MYC*) and stress-adaptive proteins even when canonical cap-dependent translation is heavily compromised [52,53]. Furthermore, this dynamic regulation facilitates the evasion of ferroptosis, an iron-dependent form of cell death. In cancers, such as acute myeloid leukemia (AML), the overexpressed ALKBH3 demethylates *ATF4* mRNA, enhancing its expression. This upregulated ATF4 shields cancer cells from toxic lipid peroxidation, conferring robust ferroptosis resistance [54]. Consequently, this m^1^A-driven translational reprogramming acts as a survival mechanism, allowing cancer cells to adapt to the hostile tumor microenvironment, evade apoptosis, and accelerate disease progression.

### 2.3. The Epitranscriptomic Crosstalk Between m^1^A and m^6^A

**Figure 1:**
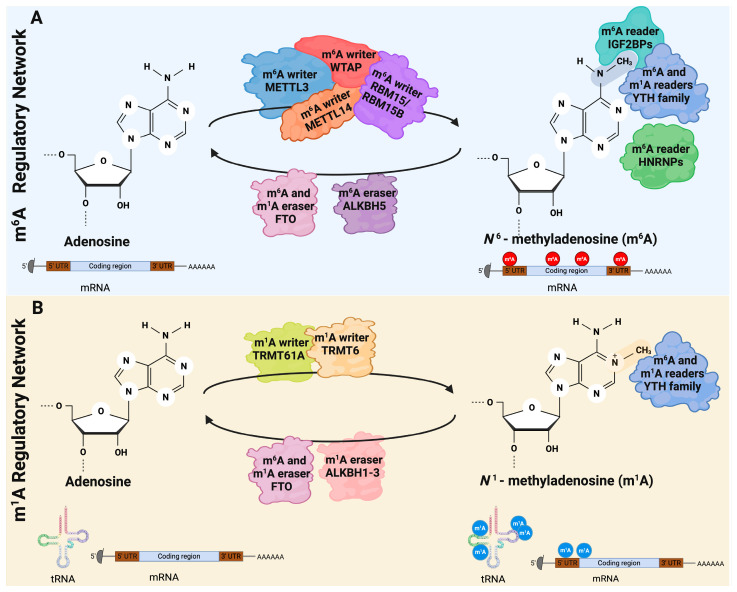
Comparative overview of m^6^A and m^1^A regulatory networks. Schematic representation of m^6^A and m^1^A regulatory proteins and the crosstalk between these modifications. (**A**) The m^6^A regulatory network. The m^6^A modification is uniquely established by the METTL3/14-WTAP-RBM15/15B writer complex and removed by ALKBH5, with transcripts recognized by diverse readers such as IGF2BPs and HNRNPs. (**B**) The m^1^A regulatory network. This network is uniquely established by the TRMT6/61A writer complex and ALKBH1-3 erasers. Despite these structural divergences, both pathways exhibit biological crosstalk mediated by shared regulatory proteins, notably the dual-eraser FTO and the dual-reader YTH family. The curved black arrows illustrate the dynamic and reversible nature of the methylation. The labeled red and blue circles represent the m^6^A and m^1^A marks on RNAs. Abbreviations: ALKBH1-3, AlkB homolog 1 to 3; ALKBH5, AlkB homolog 5; FTO, fat mass and obesity-associated protein; HNRNPs, heterogeneous nuclear ribonucleoprotein; IGF2BPs, insulin-like growth factor-2 mRNA-binding proteins; m^1^A, N^1^-methyladenosine; m^6^A, N^6^-methyladenosine; METTL3/14, methyltransferase-like 3/14; mRNA, messenger RNA; RBM15/15B, RNA-binding motif protein 15/15B; tRNA, transfer RNA; TRMT6/61A, tRNA methyltransferase 6/61; UTR, untranslated region; WTAP, Wilms tumor 1-associated protein; YTH, YT521-B homolog. Created in BioRender, v2026. Akgül Lab. Available online: https://BioRender.com/mj1zte7 (accessed on 12 June 2026). Agreement number: MS29TXQ5CC.

Although m^6^A is the most prevalent internal mRNA modification, m^1^A is distinctly characterized by a positive electrostatic charge that disrupts Watson–Crick base pairing, unlike the neutral m^6^A, which weakens but still permits base-pairing [55,56]. Furthermore, m^1^A is primarily enriched in 5’ UTRs and near start codons to regulate translation initiation, whereas m^6^A is typically localized near stop codons and 3’ UTRs [57,58]. Despite these positional differences, the two modifications are tightly linked through shared regulatory proteins, such as erasers and readers (Figure 1). The demethylase FTO, originally identified as an m^6^A eraser, also exhibits demethylase activity toward m^1^A, providing a direct intersection between different adenosine methylation pathways [32,45]. Additionally, reader proteins from the YTH domain family, such as YTHDF1, YTHDF2, YTHDF3, and YTHDC1 directly recognize and bind to m^1^A sites. For example, as in the case of m^6^A degradation, YTHDF2 can bind to m^1^A-modified transcripts and facilitate their degradation by recruiting other proteins [33]. Beyond the shared enzymatic machinery, the relationship between m^1^A and m^6^A is deeply rooted in their chemical structure. Most notably, m^1^A can undergo a chemical conversion directly into m^6^A under alkaline conditions through a process known as the Dimroth rearrangement [59]. This intrinsic structural relationship emphasizes their chemical interconnectivity and highlights how these modifications can interact dynamically, which can facilitate the detection of m^1^A [60].

Beyond chemical and enzymatic overlap, this epitranscriptomic crosstalk functionally extends to the coordinated modulation of canonical cancer signaling networks, including the phosphatidylinositol 3-kinase (PI3K)/protein kinase B (AKT), mitogen-activated protein kinase (MAPK), Wnt/β-catenin, and MYC pathways. Rather than controlling the entire cascades, epitranscriptomic marks specifically modulate their critical genes. For instance, aberrant m^6^A methylation stabilizes the transcripts encoding upstream receptors such as *EGFR* [60] or downstream effectors like *KRAS* [60], thereby driving oncogenic signaling through the PI3K/AKT and MAPK axes. Similarly, specific m^6^A marks on non-coding RNAs such as *circRNA-SORE* [60] directly sustain Wnt/β-catenin signaling to drive therapeutic resistance. Furthermore, the regulation of the *MYC* oncogene demonstrates the functional convergence of these pathways. Its oncogenic output is supported by both m^6^A-mediated transcript stabilization [60] and m^1^A-driven translational reprogramming [60] to bypass cellular stress. Ultimately, this targeted epitranscriptomic regulation over specific nodes within classical signaling cascades provides the biological rationale for developing precision inhibitors.

## 3. Therapeutic Targeting Approaches for m^6^A

### 3.1. Pharmacological Approaches: Small Molecule Inhibitors for m^6^A

Given the biological impact of m^6^A dysregulation on cancer signaling cascades, there is a clinical rationale for pharmacologically disrupting this network [39]. Historically, targeting the m^6^A machinery has presented considerable challenges, evolving from structurally non-specific pan-inhibitors to more selective catalytic blockers. These targeted small molecules primarily function by binding to the catalytic regions of the enzymes, thereby inhibiting substrate or cofactor binding through competitive or allosteric mechanisms [39]. Understanding this structural and pharmacological evalution provides insights for overcoming the pharmacokinetic barriers that currently limit the clinical translation of epitranscriptomic drugs.

#### 3.1.1. METTL3 Inhibitors

*Adenosine Analogues*

The initial efforts for exploration of pharmacological inhibitors for the epitranscriptome relied on adenosine analogues. Foundational structural analyzes established that METTL3 functions as the catalytic core of the METTL3-METTL14 complex, housing the SAM binding pocket. The physiological byproduct of methylation, S-adenosylhomocysteine (SAH), directly binds to this pocket and acts as an endogenous product inhibitor [39]. Similarly, sinefungin, a natural pan-methyltransferase inhibitor, structurally related to SAM, has been shown to occupy the catalytic site and block methyl donor transfer, thereby providing a structural basis for an inhibitor design targeting METTL3 [61]. Moreover, molecules, such as 3-Deazaadenosine [62], Neplanocin A [63], and adenosine dialdehyde [64] are examples of classical SAH hydrolase inhibitors that elevate intracellular SAH levels and thereby suppress SAM-dependent transmethylation reactions.

While essential for validating that the METTL3 SAM-binding pocket is druggable [61], adenosine analogues remain strictly preclinical tool compounds. Their clinical translation is precluded by the structural conservation of the SAM domain, which confers poor cell permeability and weak selectivity leading to broad off-target epigenetic toxicity against DNA and histone methyltransferases as well as *in vivo* toxicity [65,66,67,68]. Consequently, overcoming this severe pan-methylation toxicity has become the structural challenge driving the development of next-generation, METTL3-specific inhibitors.

*UZH1a and UZH2*

To overcome the poor selectivity of adenosine analogues, a structure-based drug design was used to develop novel METTL3 inhibitors. This has led to the discovery of UZH1a, an aminopyrimidine derivative, and its triazaspiro analogue, UZH2 [69,70]. Mechanistically, these compounds act as SAM-competitive inhibitors, but achieve high target selectivity through conformational reorganization within the METTL3 SAM binding pocket. Crystallographic studies have revealed that, upon binding, UZH compounds reorient Lysine 513 (Lys513). This structural shift, which does not occur with early pan-inhibitors, accommodates the inhibitor while preventing off-target binding to other methyltransferases [69].

Unlike adenosine analogues, UZH inhibitors readily penetrate cell membranes to suppress global m^6^A RNA methylation. They have displayed strong cellular target engagement in various cancer models, successfully reducing m^6^A levels in acute myeloid leukemia (MOLM-13), prostate cancer (PC-3), osteosarcoma (U2OS) and human embryonic kidney (HEK293T) cell lines [69,70].

Despite their high selectivity, UZH1a and UZH2 remain as preclinical chemical probes rather than drug candidates as their therapeutic usage is hindered by two primary limitations. First, their efficacy significantly drops in cellular environments due to intense competition from high intracellular SAM concentrations [69]. Second, poor pharmacokinetic properties, specifically low metabolic stability and rapid clearance, preclude their use in *in vivo* models [70]. Consequently, the need to overcome these profound pharmacokinetic barriers has led the discovery of STM2457, the first *in vivo*-active METTL3 inhibitor [71].

*STM2457*

As a foundational molecule in epitranscriptomic pharmacology, STM2457 is recognized as the first-in-class catalytic inhibitor of METTL3. Introduced by Yankova and colleagues in 2021, it provided the initial proof-of-concept that targeting the m^6^A modification machinery is a viable, targeted therapeutic strategy in preclinical *in vivo* models of AML [71]. Mechanistically, STM2457 functions as a SAM-competitive antagonist. It selectively occupies the SAM binding pocket of METTL3 enzyme. By physically obstructing this catalytic site, STM2457 prevents the transfer of the methyl donor required for m^6^A RNA modification. In oncological contexts, this pharmacological intervention induces a global depletion of m^6^A marks, which subsequently impairs the stability and translation of oncogenic mRNAs. For instance, in AML models, METTL3 inhibition by STM2457 directly suppresses the m^6^A modification of critical leukemogenic transcripts including *c-MYC*, *HOXA10*, *SP1* and *BRD4*. This resultant hypomethylation diminishes their consequent translation efficiency, thereby inducing apoptosis and driving terminal differentiation [71].

Recently, the therapeutic evaluation of STM2457 has expanded beyond hematological malignancies, demonstrating substantial anti-tumor activity across a spectrum of solid tumor models. In the context of gastric cancer, systemic administration of STM2457 disrupts tumor-intrinsic survival pathways by profoundly altering the global m^6^A modification landscape. Phenotypically, this epitranscriptomic blockade induces cell cycle arrest in the S phase and subsequent apoptosis. Consequently, STM2457 effectively suppresses gastric cancer cell proliferation and migration *in vitro* and significantly inhibits tumor growth in subcutaneous xenograft murine models *in vivo*. This establishes a robust preclinical foundation for its potential application in gastric malignancies [72]. Similarly, in colorectal cancer models, STM2457 strongly suppresses cell growth and induces apoptosis *in vitro*, while inhibiting tumorigenic capacity in preclinical murine xenograft models. Mechanistically, this anti-tumor effect is mediated through the marked downregulation of Asparagine synthetase (ASNS). STM2457 treatment decreases the m^6^A level in *ASNS* mRNA, leading to its reduced expression. This underscores the critical role of the METTL3/m^6^A/ASNS regulatory relationship in colorectal cancer progression and survival [73].

The existing oncological data indicate that STM2457 can serve as a valuable adjunct to circumvent multidrug resistance. Studies on non-small cell lung cancer demonstrate that targeting METTL3 with STM2457 significantly augments the chemosensitivity of malignant cells to cytotoxic agents, such as paclitaxel or carboplatin. This sensitization achieved by attenuating the expression of cellular efflux pump ATP binding cassette subfamily C member 2 (ABCC2) via the m^6^A-YTHDF1-dependent regulatory axis [74]. Parallel studies in ovarian cancer have established that METTL3 inhibition via STM2457 disrupts the METTL3-ADAM23 regulatory axis, thereby resensitizing aggressive malignancies to platinum-based chemotherapies *in vivo* [75].

Beyond direct tumor-intrinsic cytotoxicity, emerging evidence highlights the profound immunomodulatory advantages of STM2457 within the tumor microenvironment. Pharmacological inhibition of METTL3 by STM2457 leads to the accumulation of immunostimulatory endogenous double-stranded RNAs (dsRNAs). This accumulation triggers a robust cellular interferon response via innate immune sensors, which significantly enhances tumor antigen presentation and sensitizes previously immunosuppressed cold tumors to anti-PD1 immune checkpoint blockade. This immunogenic mechanism presents a highly promising combinational strategy for advancing cancer immunotherapy [76,77].

A primary pharmacological advantage of STM2457 is its target specificity; it exhibits a more than 1000-fold biochemical affinity for METTL3 compared to other methyltransferases, which significantly mitigates the risk of off-target epigenetic perturbations [71]. However, despite these robust preclinical achievements, STM2457 remains strictly a preclinical tool compound and is yet to advance to human clinical trials. Its clinical translation is primarily impeded by its poor oral bioavailability and rapid metabolic clearance *in vivo*. This pharmacokinetic limitation necessitates continuous or frequent dosing regimens in animal models, underscoring a fundamental requirement for further medicinal chemistry optimization to engineer metabolically stable and orally bioavailable derivatives suitable for human trials [71,78]. Ultimately, resolving these pharmacokinetic vulnerabilities catalyzed the evolution of clinical-stage inhibitors, most notably STC-15 [79,80].

*STC-15*

STC-15 is an orally bioavailable, SAM-competitive catalytic inhibitor of METTL3. Developed through the structural optimization of its predecessor STM2457, STC-15 has displayed improved metabolic stability and pharmacokinetic properties suitable for systemic oral administration. Currently, it is the first RNA-modifying enzyme inhibitor undergoing evaluation in human clinical trials for the treatment of advanced solid tumors [79,80].

STC-15 targets context-dependent oncogenic transcripts. In AML, STC-15 abrogates the m^6^A-dependent stabilization of Integrin alpha 4 (*ITGA4*) mRNA, suppressing leukemic cell homing and *in vivo* engraftment capacity [80]. Moreover, preclinical models demonstrate that STC-15-mediated METTL3 inhibition downregulates the anti-apoptotic BCL2 protein. This downregulation mechanistically primes AML cells for combinational therapy, revealing a profound synergistic effect between STC-15 and the BCL2 inhibitor venetoclax. In patient-derived xenograft models, this dual-targeted approach outperformed monotherapy, extending overall survival [81].

Preclinical studies revealed that the anti-tumor efficacy of STC-15 is linked to innate immune activation. STC-15-mediated METTL3 inhibition induces aberrant mRNA processing generating endogenous double-stranded RNAs. The cytoplasmic sensor MDA5 recognizes these dsRNAs, triggering a cell-intrinsic Type I interferon response that recruits anti-tumor T-cells into the tumor microenvironment [82].

Clinically, interim results from the ongoing Phase 1 (NCT05584111) [83] trial suggest a manageable safety profile and confirm systematic target engagement. Biomarker analyses from patient cohorts provide the proof-of-concept by validating the activation of innate immunity and Type I/II interferon responses, supporting the rationale for future combinational trials with immune checkpoint inhibitors [84]. Building upon these early results, STC-15 recently advanced into a pivotal Phase1b/2 clinical trial (NCT06975293) [84]. This study evaluates STC-15 through a dual clinical strategy. First, it acts as a targeted monotherapy for specific relapsed sarcomas (dedifferentiated liposarcoma and leiomyosarcoma). Second, it is tested in combination with the immune checkpoint inhibitor toripalimab (an anti-PD-1 antibody) in advanced solid tumors. This progression makes STC-15 the first RNA-modifying enzyme inhibitor to reach Phase 2, firmly translating preclinical proof-of-concept into definitive clinical evaluation [85].

A primary pharmacological advantage of STC-15 is its oral bioavailability combined with its dual capacity to inhibit m^6^A modification and activate innate immunity. As an early-phase investigational drug, STC-15 faces clinical limitations, including the need to determine optimal dosing regimens, identify predictive biomarkers, evaluate long-term patient survival outcomes and potential resistance mechanisms [83]. Despite these challenges, its high potential lies beyond monotherapy. By acting as an ‘epitranscriptomic primer’ that converts immunologically cold tumors into hot ones, STC-15 establishes a compelling rationale for rationally designed combinatorial trials with immune checkpoint inhibitors [82,84]. However, the deliberate induction of systemic interferon signaling requires careful evaluation. While this mechanism recruits anti-tumor T-cells, excessive activation of the innate immune system poses a risk for immune-related toxicities [86]. Additionally, prolonged interferon responses may trigger compensatory immune evasion, potentially limiting long-term therapeutic efficacy [87]. Alongside the clinical evaluation of STC-15, ongoing structural optimization efforts have led to the development of newer METTL3 inhibitors, such as EP652 and EP102 [88].

*EP652 and EP102*

EP652 is a highly selective SAM-competitive METTL3 inhibitor, structurally optimized to further overcome the clearance limitations of earlier chemical probes. Its optimized scaffold provides high complementarity to the METTL3 binding pocket, ensuring potent target inhibition while maintaining selectivity against other methyltransferases [88]. Crucially, EP652 exhibits high cellular permeability and metabolic stability. These properties have translated into a favourable pharmacokinetic profile and sustained *in vivo* target engagement [88]. Consequently, EP652 induced profound m^6^A depletion and broad anti-tumor efficacy. In animal models, it markedly reduced leukemic population in AML xenografts and significantly suppressed tumor growth across diverse solid malignancies, including lung and ovarian cancers, without inducing significant systemic toxicity [88].

Despite preclinical results, EP652 shares inherent limitations with other SAM-competitive METTL3 inhibitors, notably a vulnerability to high intracellular SAM levels. Furthermore, it only neutralizes METTL3’s catalytic activity, leaving its m^6^A-independent, oncogenic scaffolding functions intact. Consequently, EP652 remains an *in vivo* pharmacological probe rather than a clinical candidate. While it provided evidence for the therapeutic potential of sustained METTL3 inhibition, human clinical trials involved its subsequent analogue, EP102, establishing EP652 as a valuable preclinical reference [88]. Building upon this foundation, EP102 was developed as an orally bioavailable clinical candidate with an optimized pharmacokinetic profile. It is currently under evaluation in a Phase 1 first-in-human clinical trial (NCT07163325) to assess its safety, tolerability, and preliminary efficacy in patients with solid tumors [89]. Beyond targeting the m^6^A writer METTL3, the pharmacological inhibition of m^6^A demethylases represents a parallel therapeutic frontier.

#### 3.1.2. FTO Inhibitors

*Rhein*

Rhein is a natural anthraquinone compound found in *Rheum palmatum* L. It was identified via a structure-based screen, and acts as a direct competitive inhibitor of the FTO catalytic site. Notably, it bypasses the non-specific pathways of conventional inhibitors as it does not function as a 2-oxoglutarate mimic or a metal ion chelator [90]. In cellular models (SK-N-BE(2) neuroblastoma cell line clone BE(2)-C), rhein suppresses the FTO demethylase activity, leading to increased m^6^A methylation. Historically, it served as the first chemical probe to demonstrate that FTO-mediated RNA demethylation can be pharmacologically targetable [90].

Although rhein established a foundational paradigm for targeting the FTO active site without relying on generic co-factor mimicry, its utility has been hampered by weak biochemical potency and off-target toxicity. Consequently, while these liabilities preclude its clinical viability, rhein endures as a historical milestone that has catalyzed the development of subsequent FTO-targeted therapies, such as meclofenamic acid [90,91].

*Meclofenamic Acid*

Meclofenamic acid (MA), a conventional non-steroidal anti-inflammatory drug (NSAID), was identified as a specific FTO inhibitor through structure-based screening. Crystallographic analyses have revealed that MA acts as a competitive inhibitor by directly displacing m^6^A-containing nucleic acid substrates within the FTO catalytic pocket. This mechanism exhibits notable selectivity over the related demethylase ALKBH5 [91]. Since MA has poor cellular permeability, its ethyl ester prodrug, MA2, has been preferred for cellular and *in vivo* applications. In *in vitro* models, treatment with MA2 suppressed intracellular FTO demethylase activity, driving a global accumulation of m^6^A modifications in mRNA in HeLa cells [91]. Furthermore, MA2 was shown to have marked anti-tumor efficacy by inhibiting the growth and self-renewal of cancer stem cells in glioblastoma [92].

The MA and MA2 axes provided structural and mechanistic validation for selectively targeting the FTO catalytic pocket. However, their clinical potential is hindered by weak biochemical potency and inherent cyclooxygenase (COX) inhibition, which leads to profound off-target effects [91]. Consequently, while MA and MA2 hold no viability as a clinical cancer therapeutic their molecular scaffold served as a template for structure-guided optimization leading to the development of the FB23 series [93]. Alongside these structural insights, researchers discovered that certain natural oncometabolites also possess potent FTO-inhibitory properties [94].

*R-2-Hydroxyglutarate (R-2HG)*

FTO functions as an α-KG-dependent dioxygenase, making it susceptible to natural metabolic competitors. R-2HG is an endogenous oncometabolite that accumulates in cancers with mutant isocitrate dehydrogenase 1 or 2 (IDH1/2). Structurally, it acts as a direct competitor against α-KG. By directly binding to the catalytic pocket of FTO, R-2HG suppresses its m^6^A demethylase activity [94]. In AML models, R-2HG-mediated FTO inhibition leads to an increase in global m^6^A RNA modification. This hypermethylation decreases the stability of oncogenic transcripts, such as *MYC* or *CEBPA*, thereby promoting cell-cycle arrest and apoptosis in leukemic cells [94]. Although R-2HG is a naturally occurring oncometabolite rather than a synthetic pharmacological agent, its anti-leukemic activity provided the proof-of-concept that targeting the FTO/m^6^A axis is a promising therapeutic strategy [94].

While R-2HG establishes a biological framework for FTO inhibition, its direct clinical translation is hampered by its inherent role as a tumor-promoting oncometabolite in IDH1/2-mutant contexts. Thus, rather than serving as a therapeutic agent, it functions primarily as a research tool for developing targeted, synthetic FTO inhibitors, paving the way for the FB23 series [94].

*FB23 and FB23-2*

Developed through a structure-based design targeting the FTO catalytic pocket, FB23 and its cell-permeable derivative, FB23-2, function as highly potent and direct competitive inhibitors of FTO [93]. Mechanistically, in *in vivo* AML patient-derived xenografts, FB23-2 restores the global m^6^A epitranscriptome. This restoration destabilizes oncogenic transcripts such as *MYC* and *CEBPA*. Therapeutically, it suppresses proliferation and drives the terminal differentiation and apoptosis of leukemia stem cells [93].

Despite its high *in vivo* efficacy, FB23-2 currently serves as a foundational preclinical probe rather than an active clinical candidate, primarily due to the requirement for further pharmacokinetic optimization. Nevertheless, it remains a valuable chemical tool, validating FTO as a druggable vulnerability in oncology and setting the benchmark for next-generation m^6^A-targeted therapies [93]. Furthermore, modifications to this molecular scaffold later yielded the structurally optimized Dac series [95].

*Dac51 and Dac590*

Evolving from FB23 scaffold, Dac51 features a hydroxamic acid moiety that anchors the inhibitor into FTO’s active site via hydrogen bonding at Ser229. This stable binding yields sub-micromolar FTO inhibition (IC_50_ around 0.4 μM) [95]. Subsequent optimization has led to the synthesis of Dac590, an orally bioavailable tricyclic benzoic acid derivative [96]. In solid tumors, particularly in syngeneic melanoma mice, Dac51 reprograms tumor glycolysis to enhance CD8^+^ T-cell infiltration. This metabolic shift helps to overcome immune evasion and synergizes with anti-PD-L1 therapy [95]. However, systemic metabolic reprogramming requires additional caution. Global FTO blockade may disrupt the metabolic homeostasis in healthy tissues [95]. Furthermore it could paradoxically impair the metabolic fitness of circulating normal immune cells [97]. Thus, achieving a tumor-specific drug delivery is essential to minimize severe metabolic toxicities [95]. Currently, oral administration of Dac590 robustly suppresses AML progression *in vivo*. Notably, Dac590 synergizes with the hypomethylating agent decitabine (DEC), delivering a dual-epigenetic approach that circumvents DEC-induced immune resistance [96].

The Dac series overcomes key translational barriers by enabling immune sensitization (Dac51) and oral administration (Dac590). Furthermore, this versatile chemical scaffold has paved the way for next-generation modalities. For instance, another tricyclic derivative in this family, Dac85, has emerged as an ideal targeting moiety for PROTAC development [98]. While currently at the preclinical stage, the clinical advancement of the Dac series is contingent upon rigorous investigational new drug (IND)-enabling toxicology studies and extensive human safety profiling. Nevertheless, it represents a strong pharmacological foundation for future combinatiorial therapeutic strategies [95,96]. In parallel with designing these synthetic inhibitors, researchers have also explored drug repurposing strategies, leading to the identification of CS1 and CS2 [99,100].

*CS1 and CS2*

Identified via structure-based virtual screening of clinical-stage compounds, CS1 (NSC337766 or bisantrene) and CS2 (NSC368390 or brequinar) were repurposed as competitive FTO inhibitors. Historically, both molecules have established clinical profiles. Bisantrene has been evaluated in oncological trials since the 1980s as an anthracene compound [99,100], while brequinar was clinically tested as an inhibitor of pyrimidine biosynthesis [101]. Both CS1 and CS2 molecules bind to the catalytic pocket and suppress its m^6^A demethylase activity [100]. In AML models, CS1- and CS2-mediated FTO inhibition leads to the downregulation of immune checkpoint molecule *LILRB4*. This activity dualistically suppresses leukemic stem cell self-renewal and reverses tumor immune evasion, thereby sensitizing leukemic cells to T-cell cytotoxicity.

The primary advantage of CS1 and CS2 is their repurposed nature. Since they have established clinical safety profiles, their translation to FTO-targeted trials could be accelerated. However, their main limitation is inherited off-target activity, as bisantrene retains its broad activity as an anthracene compound and brequinar fundamentally blocks pyrimidine biosynthesis. Despite these non-specific liabilities, this foundational work provides a robust clinical rationale for targeting FTO-driven immune evasion in AML [100]. In addition to FTO, ALKBH5 represents the other m^6^A demethylase being investigated as a therapeutic target.

#### 3.1.3. ALKBH5 Inhibitors

*DDO-2728 and TD19*

Achieving ALKBH5 selectivity over its structural homolog FTO is a major medicinal chemistry challenge. DDO-2728 overcomes this barrier as a highly selective inhibitor that specifically avoids mimicking 2-oxoglutarate (2-OG) [102]. Conversely, TD19 employs a target covalent strategy. It irreversibly modifies specific cysteines (C100 and C267) to permanently occlude the ALKBH5 active site without disrupting FTO [103]. In AML models, DDO-2728 restores global m^6^A levels, which destabilizes the oncogenic transcript *TACC3*. This molecular event triggers cell cycle arrest and *in vivo* tumor suppression [102]. Similarly, the irreversible blockade of TD19 delivers profound cytotoxicity. This mechanism severely restricts tumor progression in both AML (MOLM13 and NB4) and glioblastoma multiforme (A172 and U87) tumor cell lines [103].

Despite their selectivity, these early-stage inhibitors face classic pharmacokinetic bottlenecks [102]. Bypassing systemic demethylase toxicities is important for safe clinical deployment. To achieve this, future designs could leverage permanent covalent strategies based on the TD19 scaffold [102]. This approach allows the development of ALKBH5-targeted PROTACs or degrader–antibody conjugates (DACs) [102]. Ultimately, this paradigm shift toward targeted degradation will improve the clinical therapeutic index. strategies to engineer

#### 3.1.4. m^6^A Reader Inhibitors

*IGF2BP1 Inhibitor: Cucurbitacin B (CuB)*

CuB is a natural compound that functions as an allosteric inhibitor of IGF2BP1. It targets KH1–KH2 domains by forming a permanent covalent bond with the Cys253 residue. This covalent binding alters the protein’s conformational architecture, profoundly impairing its ability to recognize and bind m^6^A-modified transcripts [104,105].

In hepatocellular carcinoma (HCC) and non-small cell lung cancer (NSCLC) models, CuB destabilizes critical oncogenes such as *c-MYC* and *KRAS*. Furthermore, it remodels the tumor-immune microenvironment by suppressing PD-L1 expression, thereby promoting the infiltration of CD8^+^ T-cells and macrophages to enhance anti-tumor immunity [104,105].

CuB’s dual action of mRNA destabilization and immune activation is its key therapeutic advantage. Its main limitation is the dose-dependent toxicity typical of broad natural covalent modifiers. However, optimized derivatives like compound A11 address this limitation. This approach demonstrates potent nanomolar efficacy while effectively decoupling anti-tumor activity from baseline cytotoxicity [105]. Currently, CuB and its analogues remain strictly in the preclinical stage, establishing a foundational framework for the covalent inhibition of reader proteins. Alongside IGF2BP1, YTHDF1 has also also emerged as a druggable target.

*YTHDF1 Inhibitors*

*Tegaserod*

Tegaserod is an FDA-approved drug that has been repurposed as a specific small-molecule inhibitor of YTHDF1. Tegaserod functions by directly blocking the binding interaction between YTHDF1 and m^6^A-modified mRNAs [106]. In AML models, tegaserod disrupts the YTHDF1-m^6^A interaction. This disruption inhibits the translation of *cyclin E2* oncogenic transcript. Administration of tegaserod in patient-derived AML xenograft models displayed potent antileukemic effects. It effectively reduced tumor viability and prolonged overall survival [106].

The primary pharmacological advantage of tegaserod lies in its status as a repurposed, FDA-approved drug, allowing it to bypass the early-stage drug discovery process. Its current limitation in oncology is that it remains in the preclinical evaluation phase for leukemia. Nevertheless, tegaserod provides a translational foundation, demonstrating that YTHDF1 is a viable therapeutic target for hematological malignancies [106]. Beyond repurposed agents, the YTHDF1 inhibitor landscape has expanded with dedicated molecules like SKLB-Y13 [107].

*SKLB-Y13*

SKLB-Y13 is the first potent small-molecule inhibitor designed to target the m^6^A-binding pocket of YTHDF1. Identified through structural optimization of a tetrahydrothieno[2,3-c]pyridine scaffold, it exhibits an IC_50_ of 0.76 μM. Molecular docking and site-directed mutagenesis have confirmed that SKLB-Y13 interacts uniquely with the YTHDF1-specific residues Tyr397 and Trp470. This interaction provides high selectivity over other YTH family members. In breast cancer models, this inhibitor disrupts the m^6^A-dependent interaction between YTHDF1 and target mRNAs such as *PRPF6*, thereby impairing their translation and inhibiting tumor proliferation [107].

From an m^1^A perspective, SKLB-Y13 is a significant chemical probe. YTHDF1 was previously identified as a primary reader of m^1^A modifications, which are typically found near start codons and disrupt Watson–Crick base pairing. By occupying the conserved aromatic cage of the YTH domain, SKLB-Y13 blocks the ability of YTHDF1 to interpret both m^6^A and m^1^A marks. This dual relevance is particularly important in metabolic contexts. For instance, YTHDF1 mediates the m^1^A-dependent translational repression of mitochondrial genes like *ATP5D*. Thus, SKLB-Y13 is positioned as a potential modulator of the Warburg effect in cancer cells [34,107]. Alongside specific YTHDF1 inhibitors, broader approaches have been explored, yielding pan-inhibitors like Compound N-7 [108].

*Compound N-7*

Compound N-7 was discovered through the screening of a commercial nucleoside analogue library against the YTH domain of YTHDF1. It acts as a pan-inhibitor across five YTH domains, including YTHDF1, YTHDF2, YTHDF3, YTHDC1 and YTHDC2, with IC_50_ values ranging from 30 μM to 48 μM. The molecule functions as a nucleoside mimetic that competitively occupies the conserved aromatic cage used by these readers to recognize methylated adenosine [108].

N-7 is particularly valuable for investigating the cumulative effect of a global reader blockade. Since m^1^A recognition at position 58 of tRNA and specific mRNA sites is shared across multiple YTHDF readers, N-7 allows researchers to model a total deficiency in the cell’s ability to interpret these modifications. This is highly relevant for studying how m^1^A and m^6^A marks cooperate to dictate the fate of transcripts under acute stress, where redundant reader functions might otherwise compensate for loss of a single isoform. Beyond YTHDF1 and broad-spectrum blockade, the distinct biological roles of other reader paralogs have driven the development of specific YTHDF2 inhibitors [108].

*YTHDF2 Inhibitors*

*CK-75*

CK-75 is a high-affinity phenylpyrazole derivative discovered through fluorescence polarization screening of a synthetic small-molecule library. It binds to a small hydrophobic pocket within the YTH domain of YTHDF2. Pharmacologically, CK-75 disrupts the interaction between YTHDF2 and m^6^A-modified transcripts, preventing their recruitment to processing bodies for degradation. In AML models such as K562, treatment with CK-75 induces cell cycle arrest at the G0/G1 phase and triggers apoptosis by stabilizing transcripts that normally constrain tumor growth [109]. The discovery of CK-75 has profound implications for m^1^A research, as YTHDF2 has been validated as a key reader that recognizes m^1^A-modified mRNAs to facilitate rapid transcript destabilization. While the m^6^A pathway is well-mapped, the role of YTHDF2 in clearing m^1^A marks on transcripts encoding metabolic enzymes, such as *ALDOA*, highlights a competitive axis between readers and erasers. CK-75 serves as a vital tool for epitranscriptomic stabilization, protecting m^1^A-modified mRNAs from premature decay and providing a novel strategy to overcome m^1^A-driven doxorubicin resistance in triple-negative breast cancer (TNBC) [110].

*DC-Y13-27*

DC-Y13-27 was identified as a derivative of the earlier DC-Y13 scaffold, demonstrating significant potency in blocking YTHDF2-RNA interactions. It is particularly noted for its ability to restore protein levels of FOXO3 and TIMP1, which are typically downregulated via YTHDF2-mediated mRNA decay. In breast cancer and melanoma models, treatment with DC-Y13-27 inhibits cell proliferation and increases the secretion of IL-1β by inducing pyroptosis [109,111]. Building upon these findings, a landmark study demonstrated that DC-Y13-27 markedly potentiates the antitumor efficacy of radiotherapy in colon cancer and melanoma models. This inhibition leads to the stabilization of specific m^6^A modified transcripts that trigger the release of pro-inflammatory cytokines, thereby converting immune desert tumors into responsive hot tumors with increased immune cell infiltration [110,112]. While its functional characterization is currently m^6^A-centric, DC-Y13-27 also serves as a vital tool for m^1^A research, as YTHDF2 is a primary reader that recognizes m^1^A modifications to facilitate rapid transcript destabilization [110,112]. While YTHDF2 targets progress, the therapeutic landscape of reader inhibitors further extends to nuclear compartmentalized members like YTHDC1 [113].

*YTHDC1 Inhibitors*

*EPZ-5676 (Pinometostat)*

EPZ-5676, a well-established inhibitor of histone methyltransferase DOT1L, was recently discovered, through structural screenings, to act as a potent dual inhibitor of the nuclear m^6^A reader YTHDC1. It effectively docks into the m^6^A-recognition pocket, sterically blocking the ability of YTHDC1 to bind modified transcripts [113]. In B-cell acute lymphoblastic leukemia (B-ALL) models, EPZ-5676 disrupts the interaction between YTHDC1 and m^6^A-modified *KMT2C* mRNA. This disruption destabilizes *KMT2C* transcripts, subsequently reducing global H3K4 methylation levels. The resulting epigenetic alteration attenuates the DNA damage response and induces cell cycle arrest, thereby profoundly suppressing leukemogenesis [113].

The primary pharmacological advantage of EPZ-5676 is its newly discovered dual-targeting capability against both epigenetic (DOT1L) and epitranscriptomic (YTHDC1) vulnerabilities. However, its major clinical limitations include a restrictive pharmacokinetic profile, necessitating continuous intravenous infusion, and modest anti-leukemic efficacy when used as a monotherapy. Although EPZ-5676 originally entered Phase 1 clinical trials (NCT01684150) [114] strictly as a DOT1L inhibitor in relapsed leukemia, the recent discovery of YTHDC1 affinity requires a profound retrospective reinterpretation of its clinical data [115].

From the m^6^A perspective, these historical trials are highly valuable as they inadvertently established the first human safety, tolerability, and pharmacokinetic profile for a YTHDC1-binding agent. Furthermore, the modest monotherapy efficacy observed in Phase 1 must now be interpreted as the clinical manifestation of unintentional DOT1L/YTHDC1 dual inhibition. To address these monotherapy limitations, EPZ-5676 was subsequently evaluated in Phase 2 combination trials (NCT03701295) [116] with the DNA hypomethylating agent azacitidine. While this regime was originally designed strictly to strike two classical epigenetic pathways simultaneously, we can now appreciate that it inadvertently introduces m^6^A RNA modulation into classical DNA/histone-targeted therapy. Ultimately, EPZ-5676 stands as a unique pharmacological bridge between classical epigenetics and modern m^6^A epitranscriptomics.

*YL-5092*

YL-5092 represents a notable advance in structure-based, *de novo* drug design for the YTH family. Discovered through high-throughput library screening and subsequent structural optimization, YL-5092 is an orally bioavailable inhibitor of the nuclear reader YTHDC1. It attains high binding potency (IC_50_ = 7.4 nM) by utilizing its 4-thiazole moiety to interact directly within the conserved m^6^A-binding tryptophan cage [117].

YL-5092 demonstrates high target specificity, shows minimal cross-reactivity against other m^6^A readers and a broad panel of human kinases. In AML models, YL-5092 competitively displaces YTHDC1 from its target transcripts, inducing cellular differentiation and apoptosis. Furthermore, in patient-derived xenograft models, it effectively suppresses leukemogenesis and extends overall survival while sparing normal hematopoietic stem cells [117].

The defining advantage of YL-5092 is its high selectivity and nanomolar potency, demonstrating that the notoriously shallow aromatic cage of YTH domains can be effectively targeted by small molecules [117]. However, its strictly preclinical status underscores a broad challenge within the m^6^A field: translating exceptional *in vitro* binding affinity into clinical success. Furthermore, YL-5092 highlights a fundamental conceptual limitation of classical small-molecule inhibitors. Because YTH family readers also function as massive structural scaffolding proteins, merely occupying their m^6^A-binding pocket leaves their remaining structural domains fully intact and potentially active in oncogenic protein–protein interactions. Addressing this biological limitation requires a paradigm shift, moving away from simply blocking the reader pocket to destroying the target protein, a conceptual leap that brings us to the forefront of proteolysis targeting chimeras (PROTACs).

#### 3.1.5. The Challenge of YTH Family Redundancy and Compensatory Mechanism

While the development of isoform-specific YTH inhibitors (such as SKLB-Y13 for YTHDF1 and CK-75 for YTHDF2) presents a significant pharmacological achievement, their long-term clinical efficacy is inherently challenged by the profound biological redundancy within the YTH family [107,109,110]. Current epitranscriptomic models demonstrate that cytosolic readers, particularly YTHDF1, YTHDF2, and YTHDF3, share highly conserved aromatic cages and exhibit significantly overlapping binding profiles across both the m^6^A and m^1^A transcriptomes [33].

Consequently, the isolated pharmacological inhibition or selective degradation of a single YTHDF isoform often triggers dynamic compensatory mechanisms. Particularly in highly adaptive and aggressive malignancies, when one reader is pharmacologically neutralized, the remaining paralogs can readily occupy the vacant methylated sites, as redundant reader functions can effortlessly compensate for loss of a single isoform [108]. This compensatory binding preserves the oncogenic signalling network, prevents target transcript decay, and drives acquires therapeutic resistance. This profound biological redundancy implies that achieving durable anti-tumor responses may require a strategic shift. Rather than relying solely on isoform-selective agents, addressing this “epitranscriptomic escape” will likely necessitate the deployment of rationally designed pan-YTH inhibitors (such as Compound N-7) or combinational PROTAC strategies capable of simultaneously dismantling multiple reader paralogs to completely disrupt the reading machinery [108].

### 3.2. Proteolysis-Targeting Chimeras (PROTACs)

While classical small-molecule inhibitors have established a robust foundation for targeting m^6^A regulators, they are inherently bound by the limitations of occupancy driven pharmacology. Inhibitors must maintain constant binding to relatively shallow active sites. Therefore, they fail to address the non-catalytic and structural scaffolding functions of massive epitranscriptomic proteins. To overcome these barriers, the field is experiencing a transition toward event-driven pharmacology through PROTACs [118,119]. These heterobifunctional molecules do not merely block a target pocket. Instead, they hijack the cell’s intrinsic ubiquitin-proteosome system (UPS) to tag the oncogenic m^6^A regulator (POI-protein of interest) for complete degradation. By eliminating the protein from the cell, PROTACs eradicate both its catalytic activity and its oncogenic scaffolding networks, offering a therapeutic avenue for a broad spectrum of human cancers [118,119].

#### 3.2.1. METTL3 Degraders: WD6305, ZW27941, KH12 and AF151

The conceptual transition from METTL3 inhibition to targeted degradation illustrates the evolution of precision pharmacology. First-generation PROTACs repurposed the UZH2 chemotype as a targeting moiety to co-opt the UPS, providing the foundational validation for ubiquitin-mediated METTL3 depletion [118,119]. Building upon this scaffold, subsequent studies optimized this UZH2-based architecture to improve target clearance. While early UZH2 derivatives demonstrated moderate METTL3 depletion (~50%) in AML models, [120] further refinement generated highly potent von Hippel-Lindau (VHL) E3 ligase-recruiting PROTACs, such as WD6305 and ZW27941. These advanced agents achieve near-complete METTL3 depletion with DC_50_ values of 140 nM and 130–170 nM, respectively, in AML models [121]. Furthermore, both molecules act as selective degraders of the METTL3-METTL14 heterodimer, destabilizing and co-degrading METTL14 via the UPS [121,122].

Therapeutically, while WD6305 eradicates non-enzymatic scaffolding functions and ZW27941 synergizes with standard AML therapies (cytarabine/venetoclax) [121,122], their reliance on the UZH2 chemotype restricts chemical diversity [118,119]. To address these restrictions, KH12 and AF151 emerged as alternatives. KH12 represents a potent METTL3 PROTAC that drives dose-, time-, and ubiquitin-dependent degradation of its target [123]. AF151 provides a structurally distinct alternative. Developed through four cycles of structural refinement using the established inhibitor STM2457 [123] as a foundational scaffold, AF151 incorporates a novel indole-nicotinamide chemotype coupled with a VHL-recruiting ligand [123]. This structural diversification demonstrates that METTL3 can be effectively targeted using diverse chemical architectures [123].

In AML models (MOLM-13), both KH12 and AF151 show remarkable potency, achieving highly efficient target degradation with DC_50_ values of 220 nM and 430 nM, respectively [123]. These PROTACs exert viability inhibition and differentiation-inducing effects that significantly surpass their occupancy-based predecessors. Crucially, expanding their therapeutic potential beyond hematological malignancies, KH12 has demonstrated profound efficacy in gastric cancer models, including patient-derived organoids, where the tumorigenic role of METTL3 is largely driven by its m^6^A-independent (non-catalytic) activity [123]. This specific finding highlights the distinct pharmacological advantage of PROTACs in dismantling the non-catalytic scaffolding functions of target proteins, an obstacle insurmountable by classical inhibitors.

The advantage of METTL3 PROTACs is their capacity to eradicate both the catalytic and scaffolding oncogenic functions of the target. However, clinical translation of these first-generation degraders is severely hindered by their high molecular weight and poor solubility, frequently violating traditional drug-like metrics (the rule of 5) and restricting oral bioavailability [119]. Pharmacodynamically, their therapeutic window is narrowed by the hook effect [119,124], which is a concentration-dependent saturation phenomenon. This effect has been confirmed to diminish the efficacy of molecules like ZW27941 at high micromolar concentration [122]. Furthermore, prolonged exposure carries the risk of acquired resistance via E3 ligase downregulation [118]. Consequently, despite definitive *in vitro* and *ex vivo* efficacy, all current METTL3 PROTACs remain strictly preclinical. Overcoming these translational barriers will require structural innovations, such as macrocyclic linkers, or transitioning to smaller molecular glue degraders.

#### 3.2.2. FTO Degraders: QP73 and FP54

In contrast to METTL3, targeting the primary m^6^A eraser FTO aims to therapeutically restore m^6^A levels in FTO-dependent malignancies. The structural blueprint for FTO degradation was established by QP73, a first-in-class PROTAC linking the Dac85-mediated inhibition to a Cereblon E3 ligase recruiter [98]. Expanding this repertoire, the recently developed FP54 utilizes a distinct chemical scaffold conjugated to a VHL ligand. The successful utilization of both Cereblon and VHL systems demonstrates that FTO is highly susceptible to target ubiquitination across diverse proteasomal pathways [125].

In AML models, FTO PROTACs exhibit profound cytotoxicity. QP73 derives dose- and time-dependent FTO degradation, suppressing oncogenes like *MYC* and upregulating *ASB2* [98]. Biologically, this degradation prevents the oncogenic erasure of m^6^A. For instance, FP54 restores m^6^A on key ribosome biogenesis transcripts (*MRPL36*, *LYAR*) triggering their YTHDF2-mediated decay. This effectively cripples global protein translation and induces apoptosis. Consequently, both degraders significantly reduce tumor burden and prolong survival in *in vivo* xenograft models [98,125].

While FTO PROTACs successfully disrupt the self-renewal dependency of leukemic cells, their clinical translation faces significant bottlenecks. Similar to METTL3 degraders, they violate the rule of 5, restricting them to preclinical stages. More importantly, because FTO critically regulates systemic energy homeostasis, whole-body FTO degradation poses a severe risk of metabolic and neurological on–target, off-tumor toxicities [125,126]. Overcoming this barrier will require a paradigm shift toward precision degradation. Further efforts must prioritize tumor-targeted delivery platforms, such as degrader–antibody conjugates (DACs), aptamer-PROTACs or targeted lipid nanoparticles (LNPs), to widen the therapeutic window and selectively clear leukemic FTO without disrupting healthy metabolism [127].

#### 3.2.3. Reader Degraders

Unlike METTL3 and FTO, m^6^A readers, such as YTHDF and IGF2BP families, lack deep and classical enzymatic active sites. Their biological functions rely on RNA-binding domains and intrinsically disordered regions. This structural reality makes them notoriously undruggable by conventional small-molecule moieties, severely stunting the development of reader-targeted PROTACs. To date, the selective degradation of YTHDF2 via small molecules remains elusive. Instead, successful degradation has been observed only serendipitously with multi-kinase dual-targeting PROTACs designed primarily for Aurora kinases [128]. This heavy reliance on off-target or multi-target degradation highlights the challenge of specifically disrupting reader proteins without relying on classical enzymatic inhibition.

To bypass this undruggable barrier, synthetically modified m^6^A-oligonucleotide PROTACs can be used as targeting warheads, exploiting the readers’ natural ultra-high affinity for methylated motifs. This strategy has already been successful against RNA-binding proteins, such as Lin28B, [129] and enables selective and native-like docking. While the development of targeted m^6^A-oligonucleotide-based PROTACs promises to unlock YTHDFs and IGF2BPs for use in clinical oncology, neutralizing these pathways does not strictly dictate the degradation of the reader protein itself. As a complementary strategy, Targeted RNA Degradation (TRD) platforms, such as Ribonuclease Targeting Chimeras (RIBOTACs), circumvent the druggability limits of these reader proteins by recruiting endogenous nucleases (e.g., RNase L), to directly cleave the modified oncogenic transcripts they bind [130]. Together, these protein- and RNA-centric degradation approaches represent the next leap in silencing reader-dependent networks.

## 4. Therapeutic Targeting Approaches for m^1^A

### 4.1. Pharmacological Approaches: Small Molecule Inhibitors for m^1^A

Unlike the m^6^A writer METTL3, which has successfully transitioned to clinical trials with specific small-molecule inhibitors (e.g., STC-15), targeting the m^1^A writer machinery (TRMT6/TRMT61A) remains an unmet axis in precision oncology. Building upon the paradigm established by the m^6^A field, the pharmacological targeting of the m^1^A epitranscriptome via small molecules has recently emerged as a highly promising therapeutic frontier, particularly in oncology. This approach fundamentally relies on the precise binding of small molecules to the catalytic regions or cofactor-binding sites of m^1^A regulatory enzymes such as S-adenosylmethionine (SAM)-binding pocket of writers or Fe(II)/α-ketoglutarate-dependent active site of erasers. By impeding substrate or cofactor engagement through competitive or allosteric mechanisms, these agents disrupt aberrant epitranscriptomic networks. Accordingly, targeting m^1^A regulators with specific small molecule inhibitors provides a powerful strategy for the pharmacological manipulation of translational reprogramming and cancer cell survival.

#### 4.1.1. m^1^A Writers: An Unmet Clinical Need

Inhibiting TRMT61A presents significant potential for enhancing immunotherapies [57]. The inhibition of TRMT61A directly reduces the synthesis of oncogenic MYC proteins and immune-evasion molecules, such as PD-L1 [131]. For example, when tumors are treated with oncolytic herpes simplex virus (oHSV), cancer cells reactively increase their m^1^A levels and upregulate PD-L1 expression [131,132]. Combining this treatment with TRMT61A inhibitors decreases the *de novo* synthesis of PD-L1, which effectively weakens the tumor’s immune escape mechanisms and renders it much more susceptible to immune system attacks [131,133]. Historically, the inhibition of RNA methyltransferases relied on broad-spectrum S-adenosylmethionine (SAM) competitors, such as sinefungin, which are clinically unviable due to severe pan-methylation toxicity [134,135].

*TRMT61A Inhibitor: 1,2,3,4,6-penta-O-galloyl-β-D-glucose (PGG)*

Targeting TRMT61A has emerged as a promising therapeutic strategy for halting tumor progression, for example, in colorectal cancer. In the absence of rationally designed synthetic inhibitors, recent structure-based drug discovery and screening efforts have turned natural compound libraries into potential TRMT61A modulators. A notable candidate emerging from these efforts is 1,2,3,4,6-penta-O-galloyl-β-D-glucose (PGG), a highly bioactive naturally occurring gallotannin. Current pharmacological models suggest that bulky and hydrogen-bond-rich polyphenols, such as PGG, can competitively occupy the SAM-binding pocket of TRMT61A, effectively blocking its methyltransferase activity [136].

While PGG serves as an important proof-of-concept for targeting the m^1^A writer machinery, its clinical translation requires caution. Polyphenolic compounds frequently behave as pan-assay interference compounds (PAINS). Consequently they exhibit off-target effects across various enzymes and displaying suboptimal *in vivo* bioavailability [137].

Currently, the disruption of the TRMT6/61A oncogenic axis relies almost exclusively on genetic interventions. Tools such as RNA interference (shRNA/siRNA) and CRISPR/Cas9 technologies have provided the current preclinical evidence, unequivocally validating TRMT6/61A as a potent therapeutic vulnerability in cancer models [136,138]. However, their direct clinical translation is hindered by delivery challenges and immunotoxicity.

Transitioning from these established genetic findings to future pharmacological perspectives, the recent resolution of the TRMT6/61A crystal structure has paved the way for structure-based drug design. In the absence of rationally designed synthetic inhibitors, recent *in silico* and *in vitro* screening efforts have identified natural compounds, such as PGG, as potential modulators. While PGG competitively occupies the SAM-binding pocket in current pharmacological models, it frequently behaves as a pan-assay interference compound with suboptimal *in vivo* bioavailability. Therefore, rather than an established therapeutic, PGG represents a future structural scaffold that requires extensive medicinal chemistry optimization. Furthermore, the development of PROTAC-mediated degraders against the TRMT complex represents a highly promising, yet currently speculative, frontier to completely dismantle this epitranscriptomic survival network [136,137,138]. As research strives to overcome these writer-targeted bottlenecks, parallel progress has been made in characterizing the small molecule inhibition of m^1^A erasers [139].

#### 4.1.2. m^1^A Erasers

*ALKBH3 Inhibitors*

*HUHS015 and HUHS015 Sodium Salt*

While the pharmacological targeting of m^1^A writers remains speculative, the inhibition of m^1^A erasers provides more established evidence for m^1^A-targeted therapeutics. HUHS015 is a rationally designed, synthetic compound that has been developed as a specific small-molecule inhibitor of the m^1^A eraser ALKBH3 (PCA-1). Identified through a structure-based drug design, HUHS015 functions by directly binding to the deep Fe(II)- and α-ketoglutarate-dependent catalytic pocket of ALKBH3, thereby blocking its demethylase activity on m^1^A-modified transcripts [139]. In prostate cancer and non-small cell lung cancer (NSCLC) models, HUHS015 disrupts the ALKBH3-mediated erasure of m^1^A, thereby preventing the abnormal stabilization and continuous translation of oncogenic survival transcripts. Administration of HUHS015 in preclinical *in vivo* xenograft models demonstrated potent anti-tumor effects. It effectively induced robust cell cycle arrest, reducing tumor viability, and triggering apoptosis [139]. The primary pharmacological advantage of HUHS015 lies in its high target specificity for ALKBH3, allowing it to bypass the severe pan-methylation toxicity associated with early-generation epigenetic drugs.

The primary pharmacological challenge of the original HUHS015 was its poor aqueous solubility, which resulted in insoluble material remaining at injection sites and limited bioavailability. To overcome this hurtle, researchers developed the sodium salt form of HUHS015, which dramatically improved its pharmacokinetic profile. Following subcutaneous administration in rat models, the sodium salt version increased the area under the curve by 8-fold compared to the free base form [140]. This optimized formulation demonstrated superior anti-tumor efficacy in DU145 xenograft models due to its enhanced systemic exposure. The sodium salt form serves as the standard for ongoing research into ALKBH3-mediated metabolic reprogramming, particularly in studies involving the disruption of the H3K18la-ALKBH3-HK2 feedback loop in age-related macular degeneration [141]. However, it remains in the preclinical evaluation phase and requires further pharmacokinetic optimization. Nevertheless, HUHS015 serves as a powerful translational foundation, demonstrating that targeting the m^1^A erasure mechanism is a viable therapeutic strategy for aggressive solid tumors.

*Rhein*

Rhein, a natural compound found in traditional medicinal plants, such as rhubarb, was characterized as a selective inhibitor of the AlkB family after initial identification as an FTO inhibitor [142]. Structural studies and crystal structures (PDB: 4RFR) revealed that rhein binds directly to the ALKBH3 active site by competitively occupying the nucleotide-binding pocket rather than by chelating the catalytic iron. It inhibits ALKBH3 repair of single-stranded DNA with IC_50_ around 5.3 μM [142]. It should be noted that rhein also acts as a pan-eraser blocker by suppressing ALKBH1, thereby preventing the removal of m^1^A, m^3^C, and m^5^C marks from tRNA and DNA substrates [142].

From a therapeutic perspective, rhein acts as a chemosensitizer. In human glioma cell lines, rhein treatment synergistically enhances the cytotoxicity of methyl methane sulfonate (MMS), an alkylating agent that primarily generates m^1^A and m^3^C lesions. This effect was shown to be target-specific, as the sensitization vanished upon the silencing of ALKBH1, ALKBH2, and ALKBH3. While rhein possesses broad pharmacological activities, its ability to elevate cellular m^6^A and m^1^A levels by inhibiting multiple eraser enzymes provides a natural scaffold for the design of more specific epitranscriptomic modulators targeting ALKBH1-mediated translation and ALKBH3-mediated repair networks [142,143].

*2-Hydroxyglutarate (2-HG)*

The oncometabolite D-2-Hydroxyglutarate (D-2-HG), which accumulates to high levels (up to 35 mM) in cancers harboring IDH1/2 mutations, was reported to act as a structural antagonist of α-KG for ALKBH enzymes. D-2-HG competitively inhibits the catalytic activity of ALKBH3 with an IC_50_ of approximately 3.09 mM. This inhibition prevents the direct reversal of alkylation damage, leading to an accumulation of DNA lesions in IDH-mutant cells [144]. Kinetic analysis further determined that both D-2-HG and L-2-HG enantiomers significantly inhibit ALKBH3 at pathologically relevant concentrations, with Ki values of 545 μM and 185 μM, respectively, for m^1^A repair in ssDNA. This endogenous inhibition creates a state of BRCA-ness or repair deficiency that sensitizes tumor cells to clinical alkylating agents, such as temozolomide. This discovery provided the molecular basis for why IDH-mutant glioma patients often respond better to certain chemotherapies and highlighted 2-HG as a natural, albeit pathological, modulator of m^1^A epitranscriptome [145,146].

*ALKBH1 Inhibitors*

*Compound 13h and Compound 16*

Compound 13h has been rationally designed by structural optimization of 1H-pyrazole-4-carboxylic acid derivatives. It was identified as the first potent and highly selective small-molecule inhibitor of the DNA 6mA demethylase ALKBH1. Structurally, 13h docks into the catalytic pocket of ALKBH1, as demonstrated by co-crystal structure (PDB ID: 8K62), where it forms specific interactions that block the enzyme’s ability to erase 6mA marks from DNA. Although its primary discovery focused on DNA 6mA erasure, its inhibitory profile is of profound interest for m^1^A research. ALKBH1 is a versatile eraser that targets m^1^A at position 58 of cytosolic tRNAs and various mitochondrial tRNA sites. In cellular models, 13h and its derivative, Compound 16, successfully engage ALKBH1 and modulate the intracellular levels of its methylation targets. By blocking ALKBH1, Compound 13h potentially stabilizes m^1^A-modified tRNA pools, thereby enhancing translation initiation and usage, particularly under metabolic stress or glucose deprivation. Moreover, the high specificity of 13h allows it to bypass the severe pan-methylation toxicity associated with early-generation epigenetic drugs. While its clinical translation remains unclear at this stage, Compound 13h currently serves as an established and highly reliable preclinical tool compound for exploring the biological functions of ALKBH1 and conceptualizing its therapeutic targeting in oncology [147].

#### 4.1.3. m^1^A Readers

The protein family of YTH domain-containing readers, including YTHDF1, YTHDF2, YTHDF3 and YTHDC1, serves as a convergent regulatory hub by recognizing both m^6^A and m^1^A modifications as mentioned previously. This intersection of two distinct methylation marks creates an epitranscriptomic crosstalk that allows these readers to integrate diverse RNA signals into unified biological outputs, such as coordinated control of translation and transcript stability. Consequently, targeting these reader proteins represents a high-value therapeutic strategy, as a single pharmacological agent could simultaneously disrupt multiple oncogenic pathways governed by both m^1^A and m^6^A signalling to overcome treatment resistance in “epitranscriptome-addicted” cancers. However, due to the absence of any small molecules exclusively targeting m^1^A-readers, the pharmacological exploitation of this crosstalk strictly remains a future perspective, awaiting advanced structure-based drug discovery.

## 5. Discussion, Challenges and Future Perspectives

**Figure 2:**
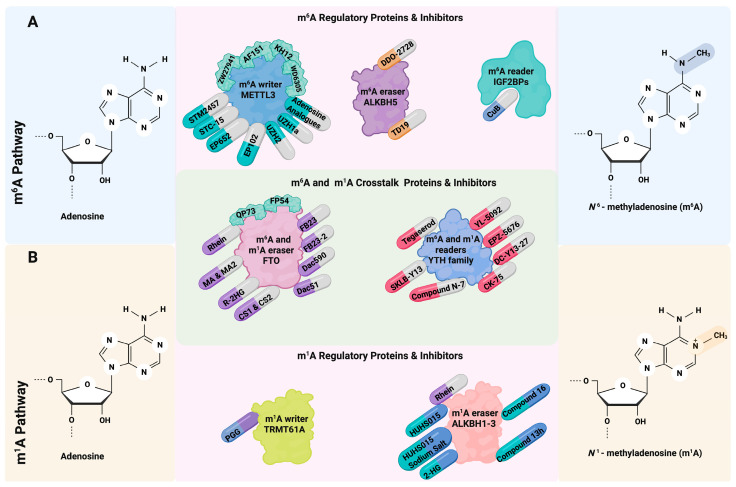
The pharmacological landscape and biological crosstalk of m^1^A and m^6^A regulatory networks. (**A**) The m^6^A-specific regulatory axis. m^6^A modification is dynamically regulated by a dedicated set of writers (e.g., METTL3), erasers (e.g., ALKBH5), and is recognized by readers (e.g., IGF2BPs). Representative small-molecule inhibitors (depicted as pill shapes, e.g., STM2457, STC-15) and PROTAC-based degraders (depicted as green shapes; e.g., WD6305, ZW27941, AF151, KH12) targeting these components are highlighted. (**B**) The m^1^A-specific regulatory axis. Analogous to the m^6^A network, the m^1^A pathway consists of specific enzymatic components, including the TRMT61A writer complex and ALKBH1-3 erasers, which are targeted by various pharmacological agents (e.g., PGG, Rhein). The central panel illustrates the mechanistic crosstalk between the two pathways, involving regulatory proteins with dual specificity or coregulatory roles, such as the eraser FTO and the YTH reader family (e.g., R-2HG, Dac51). This crosstalk is mediated by shared small-molecule inhibitors (e.g., R-2HG, Dac51) as well as FTO-targeted PROTACs (e.g., QP73, FP54). Abbreviations: ALKBH1-3, AlkB Homolog 1 to 3; ALKBH5, AlkB Homolog 5; FTO, Fat mass and obesity-associated protein; IGF2BPs, Insulin-like growth factor-2 mRNA-binding proteins; m^1^A, N^1^-methyladenosine; m^6^A, N^6^-methyladenosine; METTL3, methyltransferase-like 3; PROTACs, Proteolysis-targeting chimeras; TRMT6/61A, tRNA methyltransferase 6/61; YTH, YT521-B homolog. Created in BioRender, v2026. Akgül Lab. Available online: https://BioRender.com/hep8eep (accessed on 12 June 2026). Agreement number: WG29TXOTUN.

The clinical and preclinical evidence covered in this review underscores a significant disparity in the therapeutic maturation of m^1^A and m^6^A RNA methylation networks. To date, the robust pharmacological targeting of m^6^A has successfully established epitranscriptomics as a druggable frontier in oncology, whereas interventions targeting m^1^A remain largely in their preclinical infancy. Despite this developmental gap, the m^1^A and m^6^A regulatory pathways do not function as mutually exclusive systems. Rather, their ultimate biological output is dictated by an intricate epitranscriptomic crosstalk (Figure 2). Because these marks can be collaboratively regulated by dual-erasers (e.g., FTO) and competitively recognized by overlapping reader proteins, pan-inhibition risks unpredictable phenotypic consequences [90,98]. Therefore, future pharmacological strategies must achieve high isoform selectivity to specifically disrupt oncogenic networks while preserving tumor suppressive signalling.

Despite the clinical advancements in m^6^A targeting, the translation of epitranscriptomic modulators faces significant pharmacological, pharmacokinetic, and structural challenges (Table 1). Because these methylation networks govern fundamental physiological homeostasis, systemic pharmacological intervention carries severe on–target, off-tumor toxicity risks [126]. To mitigate systemic toxicity [126], successful clinical translation necessitates biomarker-guided patient selection to identify responsive cohorts. Rather than universal administration, it is essential to profile specific epitranscriptomic vulnerabilities. For instance, evaluating METTL3 or FTO expression levels restricts these inhibitors to relevant malignancies, minimizing adverse effects in non-responders [126]. Similarly, metabolic markers like IDH1/2 mutations identify patients with endogenously suppressed ALKBH and FTO activity, predicting their increased sensitivity to specific treatments such as alkylating agents (e.g., temozolomide) [126]. Furthermore, baseline immune signatures, such as interferon signaling, are actively utilized in clinical trials (e.g., STC-15) to predict therapeutic outcomes [126]. Concurrently, advancing these modulators through clinical pipelines presents regulatory challenges, as securing clinical approval for these small molecule inhibitors demands rigorous toxicological profiling.

While the transition toward PROTACs offers a novel strategy to degrade oncogenic scaffolding proteins, first generation degraders invariably violate rule of 5 due to their high molecular weight. Furthermore, their clinical utility is inherently hindered by the hook effect and rapid emergence of E3 ligase-mediated acquired resistance [119].

Beyond systemic toxicity, structural limitations dictate the future of targeted design. Because reader proteins rely on shallow RNA-binding grooves and intrinsically disordered regions rather than deep catalytic pockets, they have historically been considered undruggable [128]. First-generation small molecules and pan-inhibitors (such as SKLB-Y13 and CK75) serve as valuable experimental probes to decode the complex crosstalk between m^6^A and m^1^A reader networks *in vitro*. However, translating these classical inhibitors into potent, selective *in vivo* therapies remains exceptionally challenging. To bypass these systemic and structural barriers, the entire field must transition toward precision delivery platforms. Expanding the universal application of targeted vehicles (e.g., DACs and LNPs) will be crucial to optimize the pharmacological safety margin and mitigate off-tumor toxicity across all epitranscriptomic targets [127]. Concurrently, to overcome the architectural constraints of readers, such as YTHDFs and IGF2BPs, future interventions must leverage next-generation modalities such as Oligo-PROTACs, which exploit natural binding affinities to enable selective degradation [129]. As an orthogonal approach, TRD directly cleaves oncogenic transcripts via nuclease-recruiting chimers, circumventing the structural barriers of conventional protein inhibitors [130]. Furthermore, because m^6^A and m^1^A methylations uniquely alter local RNA folding landscape, [47] targeting these distinct methylations-dependent structural RNA motifs by RIBOTACs may provide a strategy for selective degradation of oncogenic RNAs [130].

To circumvent the dose-dependent toxicity of conventional pan-inhibitors, the next frontier in precision oncology relies on site-specific epitranscriptomic editing. Recent breakthroughs in CRISPR-Cas13 engineering offer programmable tools capable of modifying individual transcripts without disturbing the global methylation balance. For instance, the dm^6^ACRISPR system utilizes a catalytically dead Cas13b-ALKBH5 fusion protein to direct the targeted demethylation of oncogenic mRNAs such as *EGFR* and *MYC*, potently suppressing tumor proliferation [148]. Conversely, Targeted RNA Methylation (TRM) platforms employing dCas13-METTL3/14 fusions enable site-specific m^6^A incorporation to manipulate alternative splicing and transcript abundance [149]. Pushing spatiotemporal boundaries, optogenetic platforms (e.g., PAMEC) integrate light-inducible domains with dCas13 for remote-controlled m^6^A editing within defined cellular microenvironments [150]. Beyond CRISPR-dependent systems, CRISPR-free steric blockade strategies present significant translational potential. Proof-of-concept studies demonstrate that site-specific morpholino antisense oligonucleotides (MAOs) can physically mask exact m^6^A consensus motifs. Notably, MAO-mediated blockage of specific m^6^A sites on *circRNA-SORE* successfully reversed sorafenib resistance in hepatocellular carcinoma [151]. Ultimately, integrating these programmable RNA editors and steric blockers with advanced delivery vehicles, such as LNPs, will evolve epitrancriptomic therapeutics from broad-spectrum enzymatic inhibitors into highly specific molecular tools.

Because these regulatory pathways operate through a highly interconnected biological crosstalk, future pharmacological strategies must pivot away from broad-spectrum inhibition and strictly prioritize strict isoform selectivity. Moving forward, the successful clinical translation of epitranscriptomic modulators relies on navigating severe systemic toxicities and the structural challenges inherent to non-catalytic reader proteins. The next era of drug design must, therefore, focus on achieving strict spatial and molecular precision. By integrating targeted delivery vehicles with degradation modalities, researchers may further optimize the pharmacological safety margin.

To fully appriciate the therapeutic potential of the epitranscriptome, it is imperative to explicitly differentiate between the established findings and the future perspectives within the m^1^A landscape. The established preclinical findings are currently anchored by two main pillars: first, significant *in vivo* efficacy of the specific ALKBH3 inhibitor HUHS015 [139], and second, the equivocal genetic validation demonstrating that disrupting the TRMT6/61A axis abolishes the translation of critical oncogenes and sensitizes tumors to cellular stress [136,138]. Conversely, the future perspectives in m^1^A pharmacology encompass the ongoing efforts to transform these genetic and conceptual validations into viable clinical therapeutics. The development of specific small-molecule inhibitors for TRMT61A [136,137], the generation of ALKBH1/3-targeted PROTACs, and refinement of site-specific epitranscriptomic editing tools [148,149,150,151] currently remain in the early stages of preclinical development. Fundamentally, while m^6^A currently dominates the clinical spotlight, relying on a single modification axis is insufficient to address the adaptive translational resistance of human cancers. Therefore, future research should priotize the structural and pharmacological decoding of this m^1^A regulatory machinery. Elevating these m^1^A modulators from abiological curiosity to a co-targeted therapeutic vulnerability alongside the m^6^A network will be essential to establish a comprehensive and rational treatment strategy in precision oncology.

## 6. Conclusions

Emerging insights into RNA methylation have substantially advanced our understanding of co/post-transcriptional gene regulation, positioning epitranscriptomic networks as highly actionable therapeutic targets in clinical oncology. As discussed in this review, while the m^6^A machinery currently spearheads the targeted developmental pipeline, m^1^A networks represent an equally vital, yet largely unexploited, therapeutic frontier. Ultimately, advancing m^1^A as a co-targeted vulnerability alongside m^6^A and modulating these intersecting networks could offer a rational therapeutic approach to address the adaptive translational resistance in aggressive human malignancies.

## Figures and Tables

**Table 1:** Translational landscape of epitranscriptomic modulators targeting m^6^A and m^1^A regulatory networks. This table provides a comprehensive summary of the current preclinical and clinical development status of various pharmacological agents, including small-molecule inhibitors and PROTACs. It details the specific target enzyme, inhibitor class/agents, mechanisms of action, evaluated experimental cancer models, and clinical status. Furthermore, the main pharmacological and translational limitations associated with each inhibitor class are highlighted. Abbreviations: 2-HG, 2-Hydroxyglutarate; 2-OG, 2-oxoglutarate; α-KG, α-ketoglutarate; ALKBH1-3-5, AlkB Homolog 1-3-5; AML, acute myeloid leukemia; B-ALL, B-cell acute lymphoblastic leukemia; COX, Cyclooxygenase; FTO, Fat mass and obesity-associated protein; HCC, Hepatocellular carcinoma; IDH1/2, isocitrate dehydrogenase 1 or 2; IGF2BPs, Insulin-like growth factor-2 mRNA-binding proteins; IND, investigational new drug; m^1^A, N^1^-methyladenosine; m^6^A, N^6^-methyladenosine; MA, Meclofenamic acid; METTL3, methyltransferase-like 3; NSAID, non-steroidal anti-inflammatory drug; NSCLC, Non-small cell lung cancer; PAINS, pan-assay interference compounds; PGG, 1,2,3,4,6-penta-O-galloyl-β-D-glucose; PROTACs, Proteolysis-targeting chimeras; R-2HG, R-2-hydroxyglutarate; SAM, S-adenosylmethionine; TNBC, Triple-negative breast cancer; TRMT6/61A, tRNA methyltransferase 6/61; UPS, Ubiquitin-proteosome system; YTH, YT521-B homolog.

Target Enzyme	Inhibitor Class/Agents	Mechanism of Action	Evaluated Cancer Models	Clinical Status	Limitations	References
METTL3 (m^6^A writer)	Adenosine Analogues (Sinefungin, 3-Deazaadenosine, Neplanocin A, Adenosine dialdehyde)	Pan-methyltransferase inhibition (SAM-competitive)	AML and Osteosarcoma	Preclinical (Tool compounds)	High off-target epigenetic toxicity and poor cell permeability	[69]
METTL3 (m^6^A writer)	UZH1a, UZH2 (Aminopyrimidine derivatives)	Selective SAM-competitive inhibitors (via Lys513 conformational reorganization)	AML, Prostate, Gastric and Ovarian Cancers Preclinical (Chemical probes)	Preclinical (Chemical probes)	Low metabolic stability and rapid clearance *in vivo*	[69,70,75]
METTL3 (m^6^A writer)	STM2457 (Small Molecule)	Highly selective SAM-competitive catalytic inhibition	AML, Gastric, Colorectal, NSCLC, and Ovarian Cancers	Preclinical (Tool compounds)	Poor oral bioavailability and rapid clearance *in vivo*	[71,72,73,74,75]
METTL3 (m^6^A writer)	STC-15 (Small Molecule)	Highly selective SAM-competitive catalytic inhibition; activates innate immunity	AML, Solid Tumors, Relapsed Sarcomas	Phase1b/2 (Clinical Trials)	Needs dosing regimens, predictive biomarkers and evaluation of resistance mechanisms	[80]
METTL3 (m^6^A writer)	EP652 (Small Molecule)	Highly selective SAM-competitive catalytic inhibition	AML, Lung and Ovarian Cancers	Preclinical (*in vivo* probe)	Restricted to peripheral dosing routes; lacks oral bioavailability	[88]
METTL3 (m^6^A writer)	EP102 (Small Molecule)	Highly selective SAM-competitive catalytic inhibition	Solid Tumors	Phase1 (Clinical trials)	Pending Phase 1 safety, and efficacy profiles	[89]
FTO and ALKBH3 (Pan-ALKBH erasers)	Rhein (Natural anthraquinone)	Direct competitive occupation of the catalytic pocket, acting as non-2-OG mimetic and non-chelator to block both m^6^A and m^1^A demethylase activities	Glioma and Neuroblastoma	Preclinical (Chemosensitizing tool compound)	Weak biochemical potency and broad pharmacological pleiotropy, resulting in high off-target toxicity and lack of isoform selectivity	[90,142,143]
FTO (m^6^A and m^1^A eraser)	Meclofenamic acid (MA) and MA2 (Repurposed NSAID)	Selective competitive substrate displacement (Physically occupies the catalytic pocket)	Glioblastoma	Preclinical (Molecular scaffold)	Low biochemical potency and COX inhibition, cause off-target effects.	[95,96]
FTO (m^6^A and m^1^A eraser)	R-2-hydroxyglutarate (R-2HG) (Endogenous oncometabolite)	Natural structural competitor against α-KG in the catalytic pocket	AML	Preclinical (Endogenous probe)	Inherent tumor-promoting role in IDH1/2-mutant contexts precludes therapeutic use	[94]
FTO (m^6^A and m^1^A eraser)	FB23 and FB23-2 (Small Molecule)	Highly potent, direct competitive inhibition of the catalytic pocket	AML	Preclinical (Foundational probe)	Requires further pharmacokinetic optimization for clinical advancement	[93]
FTO (m^6^A and m^1^A eraser)	Dac51 and Dac590 (Small Molecule)	Catalytic pocket inhibition via stable hydrogen bonding, facilitating metabolic reprogramming and immune sensitization	AML and Melanoma (Solid tumors)	Preclinical (Optimized leads)	IND-enabling toxicology studies	[95,96]
FTO (m^6^A and m^1^A eraser)	CS1 (Bisantrene) and CS2 (Brequinar) (Repurposed Clinical Agents)	Competitive inhibition of the catalytic pocket, reversing immune evasion	AML	Preclinical (Repurposed clinical agents)	Inherited off-target activity (Anthracene toxicity and pyrimidine biosynthesis blocking)	[99,100,101]
ALKBH5 (m^6^A eraser)	DDO-2728 and TD19 (Small Molecules)	Selective inhibition of the catalytic pocket, via non-2-OG competitive (DDO-2728) or irreversible covalent modification (TD19)	AML and Glioblastoma	Preclinical (Selective probes)	Suboptimal pharmacokinetic profiles.	[102,103]
IGF2BP1 (m^6^A reader)	CuB (Covalent Inhibitors)	Allosteric covalent modification of KH domains, altering conformational architecture to impair m^6^A-modified transcript binding	HCC and NSCLC	Preclinical (Covalent probes)	Dose-dependent toxicity typical of broad natural covalent modifiers	[104,105]
YTHDF1 (m^6^Aand m^1^A reader)	Tegaserod (Repurposed clinical agent)	Block YTHDF1 binding to m^6^A and m^1^A modified transcripts	AML	Preclinical (Repurposed clinical agent)	Pending oncology-specific clinical validation	[106]
YTHDF1 (m^6^A and m^1^A reader)	SKLB-Y13 (Small Molecule)	Selective blockage of the binding pocket, preventing interaction with both m^6^A and m^1^A modifications	Breast cancer	Preclinical (Selective probe)	Early-stage chemical probe requiring pharmacokinetic and *in vivo* profiling	[34,107]
Pan-YTH Family (m^6^A and m^1^A reader)	Compound N-7 (Nucleoside Analogue)	Competitive nucleoside mimetic occupying the conserved binding pocket across multiple YTH isoforms causing global reader blockage	Biochemical assays (Cell-free)	Preclinical (Pan-YTH probe)	Modest biochemical potency and inherent lack of isoform selectivity	[108]
YTHDF2 (m^6^A and m^1^A reader)	CK-75 (Small Molecule)	Selective blockage of the binding pocket, preventing YTHDF2-mediated recruitment of m^6^A/m^1^A-modified transcripts to processing bodies for degradation	AML and TNBC	Preclinical (Selective probe)	Early-stage chemical probe requiring pharmacokinetic and *in vivo* profiling	[109,110]
YTHDF2 (m^6^A and m^1^A reader)	DC-Y13-27 (Small Molecule)	Selective blockage of the binding pocket, stabilizing transcripts to derive pyroptosis and immune activation	Breast cancer, Melanoma and Colon cancers	Preclinical (Radiosensitizer)	Early-stage probe requiring pharmacokinetic optimization	[109,110,111]
YTHDC1 (m^6^A and m^1^A reader)	EPZ-5676 (Pinometostat) (Repurposed clinical agent)	Steric blockade of the binding pocket, preventing reader binding to modified transcripts	B-ALL	Phase1/2 (Repurposed dual inhibition)	Pharmacokinetic constraints and clinically unverified reader efficacy	[105,106,107,109]
YTHDC1 (m^6^A and m^1^A reader	YL-5092 (Small Molecule)	Selective blockage of the binding pocket via 4-thiazole moiety, preventing reader binding to modified transcripts	AML	Preclinical (Orally bioavailable probe)	Challenges in translating *in vitro* binding affinity into clinical success	[117]
TRMT61A (m^1^A writer)	PGG (Natural gallotannin)	Competitive blockage of the SAM-binding pocket, preventing methyltransferase activity	Colorectal Cancer	Preclinical (Proof-of-concept probes)	PAINS liabilities, off-target effects and suboptimal *in vivo* bioavailability	[136,137]
ALKBH3 (m^1^A eraser)	HUHS015 (Small Molecule)	Direct binding to the catalytic pocket, blocking m^1^A demethylase activity	Prostate cancer and NSCLC	Preclinical (Selective *in vivo* probe)	Poor aqueous solubility, resulting in formulation challenges and limited *in vivo* bioavailability	[139]
ALKBH3 (m^1^A eraser)	HUHS015 Sodium Salt (Optimized salt formulation)	Direct binding to the catalytic pocket, with the salt formulation enhancing aqueous solubility and systemic bioavailability	Prostate cancer	Preclinical (Optimized *in vivo* probe)	Remains in preclinical evaluation and requires pharmacokinetic optimization for clinical translation	[140,141]
ALKBH3 (m^1^A eraser)	2-Hydroxyglutarate (2-HG) (Endogenous oncometabolite)	Competitive structural antagonism of the α-KG binding pocket, blocking m^1^A repair to induce a chemosensitizing state of BRCA-ness	IDH-mutant glioma	Endogenous modulator (Chemosensitizer)	Not a delivery therapeutic; efficacy requires massive (mM) intratumoral accumulation	[144,145,146]
ALKBH1 (m^1^A eraser)	Compound 13h and Compound 16 (Pyrazole derivatives)	Selective occupation of the catalytic pocket, preventing ALKBH1-meditated demethylation and stabilizing modified tRNA pool	Pan-cancer *in vitro* models	Preclinical (Selective tool compounds)	Currently limited to *in vitro* utility; requires comprehensive *in vivo* pharmacokinetic profiling	[147]
METTL3 (m^6^A writer)	WD6305, ZW27941, KH12 and AF151 (PROTAC Degraders)	Heterobifunctional recruitment of the UPS, driving complete proteasomal degradation to eradicate scaffolding functions	AML and Gastric cancer (Including organoids)	Preclinical (First-generation degraders)	Violates rule of 5; limited by the hook effect and E3 ligase downregulation.	[118,119,123,124]
FTO (m^6^A and m^1^A eraser)	QP73 and FP54 (PROTAC Degraders)	Heterobifunctional recruitment of the UPS, driving complete proteasomal degradation to restore m^6^A and m^1^A levels and abrogate oncogenic translation	AML	Preclinical (First-generation degraders)	Violates rule of 5; severe metabolic/neurological on–target, off-tumor toxicity risks, necessitating precision delivery platforms.	[98,126,127]
YTHDFs and IGF2BPs (m^6^A and m^1^A readers)	Oligonucleotide-based PROTACs (Conceptual Paradigm)	Targeted recruitment of the UPS by exploiting the readers’ native RNA-binding affinity via synthetically modified m^6^A-oligonucleotide warheads	Pan-cancer *in vitro* models	Early Discovery (Conceptual)	True selective degradation remains elusive; it demands complex oligonucleotide delivery.	[127,129]

## Data Availability

The original contributions presented in this study are included in the article. Further inquiries can be directed to the corresponding author.

## Abbreviations

The following abbreviations are used in this manuscript:

3’ UTRs	3’ Untranslated regions
A-to-I	Adenosine to Inosine
ABCC2	ATP binding cassette subfamily C member 2
ac^4^C	N^4^-acetylcytidine
ALKBH	AlkB Homolog
AML	Acute Myeloid leukemia
ASNS	Asparagine synthetase
B-ALL	B-cell acute lymphoblastic leukemia
COX	Cyclooxygenase
CuB	Cucurbitacin B
D-2-HG	D-2-Hydroxyglutarate
DACs	Degrader–antibody conjugates
DEC	Decitabine
DPPW	Asp-Pro-Pro-Trp motif
dsRNAs	Double-stranded RNAs
EMT	Epithelial–mesenchymal transition
Fe(II)	Iron(II)
FT-IR	Fourier-transform infrared
FTO	Fat mass and obesity-associated protein
HCC	Hepatocellular carcinoma
HNRNP	Heterogeneous nuclear ribonucleoprotein
IDH1/2	Isocitrate dDehydrogenase 1 or 2
IGF2BP	Insulin-like growth factor-2 mRNA-binding proteins
ITGA4	Integrin alpha 4
L-2-HG	L-2-Hydroxyglutarate
LNPs	Lipid nanoparticles
m^1^A	N^1^-methyladenosine
m^5^C	5-methylcytosine
m^6^A	N^6^-methyladenosine
MA	Meclofenamic acid
MAOs	Morpholino antisense oligonucleotides
METTL14	Methyltransferase-like 14
METTL3	Methyltransferase-like 3
MMS	Methyl methane sulfonate
NSCLC	Non-small cell lung cancer
oHSV	Oncolytic herpes simplex virus
PAINS	pan-assay interference compounds
PGG	1,2,3,4,6-penta-O-galloyl-β-D-glucose
POI	Protein of interest
PPI	Protein–-protein interaction
PROTACs	Proteolysis-targeting chimeras
R-2HG	R-2-hydroxyglutarate
RBM15	RNA-binding motif protein 15
RIBOTACs	Ribonuclease Targeting Chimeras
SAH	*S*-adenosylhomocysteine
SAM	S-adenosylmethionine
ssDNA	Single-stranded DNA
TNBC	Triple-negative breast cancer
TRD	Targeted RNA Degradation
TRM	Targeted RNA Methylation
tRNAi-Met	Initiator methionyl-tRNA
UPS	Ubiquitin-proteosome system
VHL	Von Hippel-Lindau
VIRMA	Vir-like m^6^A methyltransferase-associated
WTAP	Wilms tumor 1-associated protein
YTH	YT521-B homolog
